# System Design of Integrated Intelligent Platform of National Fitness Based on K-Means Clustering

**DOI:** 10.1155/2022/2171553

**Published:** 2022-09-10

**Authors:** Pingping Song, Cheng Ma

**Affiliations:** ^1^TianJin University of Sport, Tianjin, China; ^2^Kedi (Tianjin) Decoration Engineering Co. Ltd, Tianjin, China

## Abstract

Voice communication is the most common, direct, and effective method of information exchange between humans. Dependent speech signal processing will inevitably become an important carrier for the interaction between people and the interaction between people and computers. With the development of science and technology, data mining has become a means for users to extract effective information from a large amount of data, and many branches have been derived. Among them, K-means clustering algorithm is used as a classic clustering analysis algorithm. It is fast and simple, and it is also affected by the randomness of the initial center selection and the interference of outliers, which may cause poor clustering, but even if the above problems exist, it does not affect its wide application in various industries. This paper applies HBase storage technology and microservice framework to the fitness system and implements a national fitness system based on HBase and microservices. The system uses HBase to store fitness information, venue opening, and usage information for national fitness people; simulation results show that the accuracy rate on the data set has an obvious improvement. A fitness system that combines massive data storage and microservice architecture can improve the utilization of fitness resources, solve the problem of fitness resources, improve professional fitness levels, and provide support for the masses who regularly exercise scientifically.

## 1. Introduction

Voice signal processing has developed rapidly and has been widely used in recent decades. However, with the rise of communication networks, artificial intelligence, smart cities, and other applications, the demand for various voice services is increasing [1]. That is to put forward higher requirements for the reliability and robustness of the sound signal processing system. However, noise interferes with the sound signal processing system in actual applications [2]. In a system environment affected by noise, the accuracy of speech recognition is significantly reduced, especially when the signal and noise are low, the ideal effect cannot be achieved in practical applications [3]. Regarding the current sound signal processing technology, the sound detection effect is ideal in a weak noise environment, but the detection performance drops sharply in a strong noise environment [4]. Therefore, the sound signal detection under the condition of low signal-to-noise ratio is still a subject that needs further research [5]. Considering the problems in the K-means algorithm, many scholars have proposed some solutions [6].

National fitness refers to the people of the whole country, regardless of men, women, young, or old. All people increase their strength and flexibility, increase endurance, and improve the ability to coordinate and control all parts of the body, thereby making the people healthy [7]. However, as the number of users continues to increase, the amount of data in the fitness system is also increasing. Traditional relational databases can no longer meet the current data storage, and data reading and writing have become the bottleneck of the fitness system [8]. Although the current Internet uses the solution of subdatabase and table to slice data, no matter from the division of business model or the management of subdatabase and table rules, the business code is coupled with the code stored in the system [9]. As a result of the continuous increase in the number of users and the rapid increase in the amount of system code, there is an urgent need for an architectural idea to split the entire large project into small modules, and the modules are isolated from each other to ease the new functions of the project [10]. Based on the above background and current situation, the research on the nationwide fitness system that combines HBase data storage and microservice architecture is of great significance to the performance improvement and application of the fitness system. Using the microservice architecture, the business requirements of the fitness system are modularized and separated, and the fitness system is decoupled from the business level, which makes the various modules of the fitness system more flexible and improves the scalability of the fitness system.

## 2. Related Work

In order to solve the sensitivity problem of selecting the initial center point in the K-means clustering algorithm, based on the density concept, the high-density area is reasonably adjusted by calculating the density parameter, and the clustering iterative method is used to select [11]. The highest density point in the high-density area is the initial point, the point farthest from the initial point is the second point, and the first two points are the central cluster to repeatedly obtain two centers of gravity [12]. Taking the two points farthest from the center of gravity as the third point, the experimental results show that the proposed algorithm can achieve higher accuracy, fewer iterations, and good clustering results. According to the literature survey, the adaptive threshold end-point detection algorithm can effectively detect the end-points between the sound segment and the nonsound segment, including stable noise and nonstationary noise with different signal-to-noise ratios. In addition, the accuracy and robustness of the algorithm are significantly better than traditional end-point detection algorithms [13]. In addition, the adaptive subband spectral entropy speech enhancement algorithm can achieve better performance under the influence of various actual mixed noise sources. In addition, it has a great effect on improving the quality of sound signals.

The literature has developed a series of nationwide fitness systems based on HBase and microservices and analyze the requirements of the fitness system and remove the requirements of the three major business modules, namely user module requirements, venue module requirements, and route module requirements [14]. According to the results of the analysis of system requirements, business users, websites, and course modules have detailed designs. At the same time, using the creativity of microservice architecture, the overall architecture of the fitness system was designed.

The literature advocates building and improving a perfect evaluation system based on a big data platform by analyzing the physical strength of personal coaches, fitness facilities, the “escape” of the gym, and the “cognition” of consumers [15] and strengthens the gym management system and improves the certification standards of fitness coaches.

The literature builds a nationwide fitness system based on a computer network platform and builds a general system module based on system demand analysis. It can be seen from the system use case diagram that the role of the system mainly includes users and managers [16]. The smart client used on this system platform can meet a variety of needs. After testing the system, the system can be run on multiple terminals. This research shows that the system constructed by this research is practical and can provide reference for follow-up-related research.

The literature focuses on the research and improvement of sound emphasis technology based on amplitude spectrum speculation [17]. Aiming at the sound emphasis problem based on the amplitude spectrum estimation theory, this article introduces the basic principles and performance of several different short-term amplitude spectrum estimation algorithms in detail and simply analyzes the advantages and disadvantages of the short-time amplitude spectrum estimation algorithm.

The literature proposes that the fitness public service system is not yet complete and lags behind other regions, the community sports facilities are outdated, in short supply, the number of fitness people is insufficient, the fitness content form is single, and the fitness public service management system is not yet complete. On this basis, the “Internet +” model researches innovative models of the community residents' fitness service system and platform in Yuyang District, continuously improves the national fitness service system, and promotes the development of the local community's national fitness service system.

## 3. Voice Quality Detection and K-Means Clustering Model Design

### 3.1. Voice Quality Detection

The Fast Fourier Transform is performed by the Fast Fourier Transform of each frame of the preprocessed audio signal, and the signal is changed from time domain data to frequency domain data.

(1) $$\begin{array}{c} X\left(i,k\right)=FFT\left[x_{i}\left(m\right)\right]\text{.} \end{array}$$

k represents the kth spectral line in the frequency domain.

Calculating the spectral line energy, the spectral line energy of each frame signal after FFT is as follows:

(2) $$\begin{array}{c} E\left(i,k\right)={\left[X\left(i,k\right)\right]}^{2}. \end{array}$$

Calculate the energy passing through the Mel filter:

(3) $$\begin{array}{c} S\left(i,m\right)=\overset{N-1}{\underset{k=0}{\sum }}E\left(i,k\right)H_{m}\left(k\right)\text{,}0\leq m\leq M\text{.} \end{array}$$

Calculate the discrete cosine transform curve. The FFT cepstrum of the sequence *x* (*n*) is

(4) $$\begin{array}{c} \hat{x}\left(n\right)=FT^{-1}\left[\hat{X}\left(k\right)\right]\text{.} \end{array}$$

The DCT of the sequence *x* (*n*) is

(5) $$\begin{array}{c} X\left(k\right)=\sqrt{\frac{2}{N}}\overset{N-1}{\underset{n=0}{\sum }}C\left(k\right)\cdot x\left(C\left(k\right)\right)\cdot \cos\left[\frac{\pi \left(2n+1\right)}{2N}\right],k=0,1,\ldots ,N-1\text{.} \end{array}$$

When the parameter N is the length of the sequence *x* (*n*), *C* (*k*) is the orthogonal coefficient, which can be expressed as follows:

(6) $$\begin{array}{c} C\left(k\right)=\left\{\begin{array}{cc} \frac{\sqrt{2}}{2}\text{,} & k=0\text{,}\\ 1\text{,} & k=1,2,\ldots ,N-1\text{.} \end{array}\right. \end{array}$$

After taking the logarithm of the energy of the Mel filter, calculate the DCT as

(7) $$\begin{array}{c} mfcc\left(i,n\right)=\sqrt{\frac{2}{N}}\overset{M-1}{\underset{m=0}{\sum }}\log\;\left[S\left(i,m\right)\right]\cos\left[\frac{\pi m\left(2m-1\right)}{2N}\right]\text{.} \end{array}$$

After the sound signal is processed to frame, the sound signal of frame I is xi, the cepstrum coefficient of MFCC is mc, and the cepstrum distance of MFCC is as follows:

(8) $$\begin{array}{c} d_{mfcc}\left(i\right)=\sqrt{\overset{p}{\underset{n=i}{\sum }}{\left(mc_{1}^{i}\left(n\right)-mc_{2}^{i}\left(n\right)\right)}^{2}}\text{.} \end{array}$$

The MFCC framing distance measurement method is based on the MFCC framing distance trajectory of each signal frame and noise frame, using MFCC parameters to detect and judge end-point detection. The MFCC parameter emphasizes the low-frequency information of the signal. Therefore, the interference of the sound signal is shielded to a certain extent. Especially in the case of channel noise and spectral distortion, the accuracy of MFCC will become higher. However, the disadvantage of this algorithm is that the process of extracting MFCC parameters is complicated, the amount of calculation is large, and it takes time.

If the time series of the sound signal is listed as *x* (*n*), preprocessed, and framed, the resulting signal is expressed as the frequency domain form of the sound signal after xi through the discrete Fourier transform.

(9) $$\begin{array}{c} X\left(k\right)=\overset{N-1}{\underset{n=0}{\sum }}x_{i}\left(m\right)\cdot e^{j2\pi mk/N},k=0,1,\ldots ,N-1\text{.} \end{array}$$

Here, N represents the frame length.

Next, the amplitude and phase angle of each component of the audio signal after DFT conversion are calculated. Here, the amplitude is as follows, and the phase angle is expressed by the following formula:

(10) $$\begin{array}{c} X_{angle}^{i}\left(k\right)=\arctan\left[\frac{\text{Im}\left(X_{i}\left(k\right)\right)}{\text{Re}\left(X_{i}\left(k\right)\right)}\right]\text{.} \end{array}$$

IS is used to represent the nonsound segment at the beginning of the sound signal, that is, the noise segment. The total number of frames of the corresponding signal can be represented by NIS, which can represent the average energy value of the noise.

(11) $$\begin{array}{c} D\left(k\right)=\frac{1}{NIS}\overset{NIS}{\underset{i=1}{\sum }}{\left|X_{i}\left(k\right)\right|}^{2}\text{.} \end{array}$$

The spectral subtraction algorithm is

(12) $$\begin{array}{c} {\left|{\hat{X}}_{i}\left(k\right)\right|}^{2}=\left\{\begin{array}{cc} {\left|{\hat{X}}_{i}\left(k\right)\right|}^{2}-\alpha \times D\left(k\right), & {\left|X_{i}\left(k\right)\right|}^{2}\geq \alpha \times D\left(k\right),\\ \beta \times D\left(k\right), & {\left|X_{i}\left(k\right)\right|}^{2}<\alpha \times D\left(k\right). \end{array}\right. \end{array}$$

**Figure 1:**
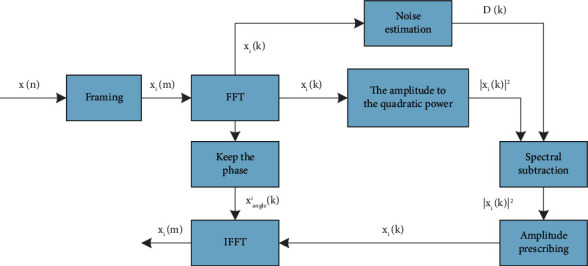
Schematic diagram of basic spectral subtraction.

The distance of the basic spectrum subtraction is shown in Figure 1.

The Vienna filter is a sound emphasis method that reproduces the sound signal from the noisy sound as much as possible. Regardless of whether the stable random process of the audio signal is continuous or discrete, it has a wide range of adaptability.

Perform windowing and frame rate signal phase spectrum through FFT, as well as further power calculation:

(13) $$\begin{array}{c} P_{y}\left(k,i\right)={\left|X_{i}\left(k\right)\right|}^{2}\text{.} \end{array}$$

Knowing that the preamble segment (noise) occupies the NIS frame, the average noise power spectrum and the average noise amplitude spectrum can be calculated as follows:

(14) $$\begin{array}{c} P_{n}\left(k\right)=\frac{1}{NIS}\overset{NIS}{\underset{i=1}{\sum }}P_{y}\left(k,i\right)=\lambda_{d}\left(k\right)\text{,}\\ {\bar{X}}_{n}\left(k,i\right)=\frac{1}{NIS}\overset{NIS}{\underset{i=1}{\sum }}\left|X_{i}\left(k\right)\right|\text{.} \end{array}$$

The voice signal combines the amplitude spectrum function with the stepped phase spectrum and restores it to the time domain through IFFT to obtain a noise-reduced sound signal:

(15) $$\begin{array}{c} {\hat{x}}_{i}\left(m\right)=IDFT\left[{\hat{S}}_{i}\left(k\right)\cdot e^{j\theta_{i}\left(k\right)}\right]\text{.} \end{array}$$

In this chapter, the algorithm introduces a short-term signal-to-noise ratio to design an adaptive threshold and uses adaptive decision to detect voice end-points. At the same time, F1 measurement is selected to evaluate the performance of the three voice end-point detection algorithms. Unlike traditional end-point detection and evaluation systems, F1 measurement can avoid the complexity of certain algorithms in the evaluation of multisyllable sentences under the condition of low signal-to-noise ratio. According to the accuracy and coverage of the measurement algorithm performance, the higher the F1 value, the higher the end-point detection effect of the algorithm. Accuracy is the ratio of the number of sound frames to the total number of sound frames with correct detection results, which measures the accuracy of the algorithm. Coverage refers to detecting the ratio of the total number of target frames to the total number of audio frames, and measuring the recall of the algorithm.

Table 1 shows the results of all F1 measurements in this experiment. The double threshold determination method, the frame distance method, and the adaptive algorithm are, respectively, suitable for sound signals with different signal-to-noise ratios and different noise environments. The signal is obtained from the NOIZEUS database, and the final experimental result is obtained by averaging F1 measurements.

### 3.2. K-Means Clustering

Use the sum of squared errors as the judgment condition:

(16) $$\begin{array}{c} SE=\overset{M}{\underset{i=1}{\sum }}\overset{m}{\underset{j=1}{\sum }}{\left|P_{ij}-C_{i}\right|}^{2}, \end{array}$$

where *M* represents the number of known clusters, *m* represents the total number of samples in each cluster, *i* in *P*_*ij*_ represents *i* clusters, *j* represents the j-th sample in the cluster, and Ci represents the centroid of the i-th cluster.

The attributes are squared, and a new attribute table is obtained after expansion, which captures the internal coupling of continuous attributes and the external coupling interaction between attributes, and calculates the Pearson coefficient, as shown in the following formula:

(17) $$\begin{array}{c} COR\left(a_{i},a_{j}\right)=\frac{\sum_{u\in L}\left(f_{i}\left(u\right)-\mu_{i}\right)\left(f_{j}\left(u\right)-\mu_{j}\right)}{\sqrt{\sum_{u\in L}{\left(f_{i}\left(u\right)-\mu_{i}\right)}^{2}}\sqrt{\sum_{u\in L}{\left(f_{j}\left(u\right)-\mu_{j}\right)}^{2}}}. \end{array}$$

Consider the hypothesis that the *p*-value test attributes are not correlated. The modified Pearson correlation coefficient is as in the following formula:

(18) $$\begin{array}{c} R\_COR\left(a_{i},a_{j}\right)=\left\{\begin{array}{cc} COR\left(a_{i},a_{j}\right)\text{,} & p\_value<0.05\text{,}\\ 0\text{,} & p\_value\geq 0.05\text{.} \end{array}\right. \end{array}$$

Calculation method of internal coupling properties:

(19) $$\begin{array}{c} RTa\left(a_{j}\right)=\left(\begin{array}{cccc} \varphi_{11}\left(j\right) & \varphi_{12}\left(j\right) & \ldots & \varphi_{1L}\left(j\right)\\ \varphi_{21}\left(j\right) & \varphi_{22}\left(j\right) & \ldots & \varphi_{2L}\left(j\right)\\ \ldots & \ldots & \ldots & \ldots \\ \varphi_{L1}\left(j\right) & \varphi_{L2}\left(j\right) & \ldots & \varphi_{LL}\left(j\right) \end{array}\right)\text{,} \end{array}$$

where *φ*_*mn*_(*j*)=*R*_*COR*(〈*a*_*j*_〉^*m*^, 〈*a*_*j*_〉^*n*^) is the Pearson correlation coefficient, where 〈*a*_*j*_〉^*m*^和〈*a*_*j*_〉^*n*^ represents the m-th power and n-th power of the *a*_*j*_ attribute column, respectively.

Calculation method of outer coupling property:

(20) $$\begin{array}{c} RTe\left(a_{j}|{\left\{a_{i}\right\}}_{i\neq j}\right)=\left(\begin{array}{ccccccc} \delta_{11}\left(j|i_{1}\right) & \ldots & \delta_{1L}\left(j|i_{1}\right) & \ldots & \delta_{11}\left(j|i_{n-1}\right) & \ldots & \delta_{1L}\left(j|i_{n-1}\right)\\ \delta_{21}\left(j|i_{1}\right) & \ldots & \delta_{2L}\left(j|i_{1}\right) & \ldots & \delta_{21}\left(j|i_{n-1}\right) & \ldots & \delta_{2L}\left(j|i_{n-1}\right)\\ \ldots & \ldots & \ldots & \ldots & \ldots & \ldots & \ldots \\ \delta_{L1}\left(j|i_{1}\right) & \ldots & \delta_{LL}\left(j|i_{1}\right) & \ldots & \delta_{L1}\left(j|i_{n-1}\right) & \ldots & \delta_{LL}\left(j|i_{n-1}\right) \end{array}\right)\text{.} \end{array}$$

Coupling representation of objects:

(21) $$\begin{array}{c} UC\left(a_{j}|A,F\right)=u\left(a_{j}\right)⊕\lambda ⊗{\left[RIa\left(a_{j}\right)\right]}^{T}+u\left({\left\{a_{j}\right\}}_{i\neq j}\right)⊕\underset{n-1}{\underset{︸}{\left[\lambda ,\lambda ,\ldots ,\lambda \right]}}⊗{\left[RIe\left(a_{j}|{\left\{a_{j}\right\}}_{i\neq j}\right)\right]}^{T}\text{.} \end{array}$$

Calculate the density parameter, Euclidean distance:

(22) $$\begin{array}{c} di\;s\tan\;ce\left[x,y\right]=\sqrt{{\left(x_{1}-y_{1}\right)}^{2}+{\left(x_{2}-y_{2}\right)}^{2}+\cdots +{\left(x_{L}-y_{L}\right)}^{2}}\text{,}\\ \begin{array}{c} MIDU\left(X_{i}\right)=\\ \overset{L}{\underset{1}{\sum }}count\left(di\;s\tan\;ce\left(x_{i},x_{j}\right)\leq \alpha *mean\_D1\right)\\ \left(0<\alpha <1\right). \end{array} \end{array}$$

Get the density of the first point MIDU, repeat the steps to get the density of all points, where *i* takes (1, L), put the density parameter in, and then calculate the mean value of MIDU, as in the following formula:

(23) $$\begin{array}{c} AvgMIDU=\frac{\overset{L}{\underset{i=1}{\sum }}MIDU\left(X_{i}\right)}{L}. \end{array}$$

Create an *E* set to place samples that meet a certain multiple of AvgMIDU. These samples form the point set *E* in the high-density area, as in the following formula:

(24) $$\begin{array}{c} E\left(x_{i}\right)>\beta \times MIDU\left(x_{i}\right)\left(0<\beta <1\right). \end{array}$$

Take two points as the center and then generate two clusters according to the distance between the center and other points. First find the cluster centroids C1 and C2 of the two clusters to find the distance to the two cluster center points in the high-density field the farthest point of is set as the third initial cluster center point, as shown in the following formula:

(25) $$\begin{array}{c} d_{i}=di\;s\tan\;ce\left(x_{i},C1\right)+di\;s\tan\;ce\left(x_{i},C2\right)\\ i\in \left(1,m\right), \end{array}$$

where *m* represents the number of sample points in the high-density area.

The data set used in the experiment: this paper uses the Parkinsons, Blood, Planning, and Vertebal data sets in UCI, as shown in Table 2.

**Figure 2:**
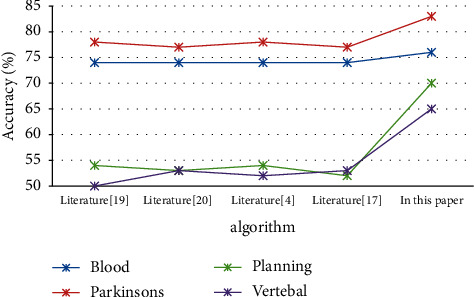
Comparison of accuracy rates under nonindependent and identical distribution.

**Figure 3:**
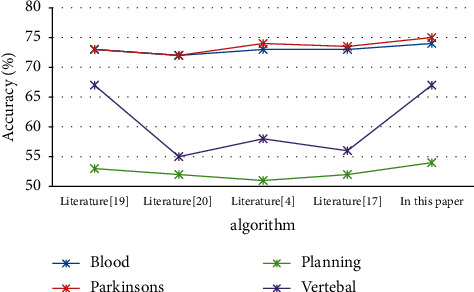
Comparison of accuracy under independent and identical distribution.

As shown in Figure 2, the parameters in the QC-Kmeans algorithm of this article are set to *α* = 0.4*β* = 0.9. This article will use the method of the next power of the nonunique distribution to process the data set and continue to use the method proposed in the literature. By comparison, it is found that the accuracy of the second power algorithm under nonindependence is increased by about 3.1% compared with other literature algorithms and reflects the superiority of the second power under nonindependent and identical distribution. In addition, it is obvious that the accuracy of each data set under independent and identical distribution has a corresponding increase of 3%, 1.6%, 1.6%, 1.7%, and 1.5% compared with independent and identical distribution, which reflects the superiority of nonindependent and identical distribution. In addition, under independent and identical distribution, *α* = 0.9*β* = 0.4 increased by 1.7%, 1.7%, 1.8%, and 1.6%, respectively. This shows that the clustering iteration method in this paper has an advantage over the optimized Min_max method in the literature, the MIN_max method in the literature, the MSDCC method in the literature, and the maximum distance product method in the literature on the Blood data set. It shows that this article the method is feasible.  Table 3 shows the experimental results on the Blood data set.  Figure 3 shows in the Comparison of accuracy under independent and identical distribution.

In Figure 2, it can be seen that the clustering iterative algorithm of second power coupling under nonindependent and identical distribution is compared with the method used in the literature of nonindependent and identical distribution under first power coupling. There are data sets in Parkinsons, Blood, Planning, and Vertebal. Obviously, this improvement is mainly due to the fact that the coupling of the second power of this article can better dig out the potential relationship between the data. It can be seen from Figure 3 that in the independent case, the clustering of this article compared with the methods in the literature, the iterative algorithm has an obvious improvement in accuracy on the Blood, Parkinson's, Planning, and Vertebal data sets. This benefits from the effectiveness and rationality of the algorithm in this paper.

## 4. Design and Practical Application of an Integrated Intelligent Platform for National Fitness

### 4.1. Demand Analysis of an Integrated Intelligent Platform for National Fitness

Ordinary users have functions such as site reservation, course selection, viewing the information of the reserved venue, and viewing the selected fitness course.

1. Venue list: ordinary users can view the list of all unexpired venues through the system homepage.
2. Course list: ordinary users can view the list of all unexpired courses through the system homepage.
3. Reservation of venues: when ordinary users find suitable venues through the venue list, they can make reservations for a certain period of time. When booking venues, they can also choose a coach for fitness guidance.
4. Order courses: ordinary users find courses that they are interested in through the course list and then order the courses.
5. List of ordered courses: ordinary users can view the course records they have ordered through the system homepage.
6. List of reserved venues: ordinary users can view the records of the venues they have reserved through the system homepage.

Venue administrators are responsible for disclosing website information to users in the system based on actual free time, allowing users to make appointments according to their needs, making the most of the free time of the website, and providing more information. For sports opportunities for people in difficulty,

1. Personal information management: after the site administrator logs into the system, he fills in personal information and uploads avatars in the personal information management.
2. Release venue: the venue administrator can release the venue in the system, fill in the venue's profile, address, type, time, and other information to release the venue.
3. List of published venues: the venue administrator can view the list of published venues through the system homepage.

### 4.2. Platform Architecture Design

**Figure 4:**
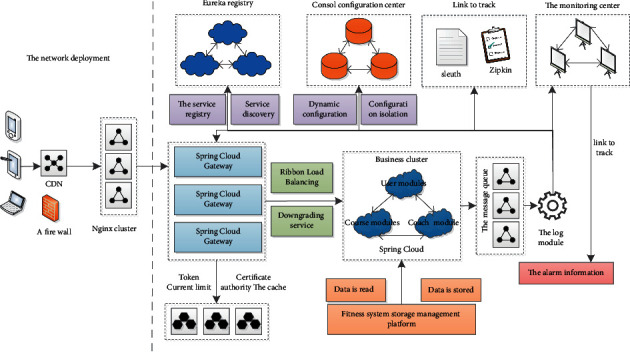
Software service architecture diagram of the National Fitness System.

The modules of the software service architecture of the national fitness system based on HBase storage and microservice architecture are interdependent and independent. As shown in Figure 4.

As shown in Figure 4, when the fitness system is split into small modules such as user modules, venue modules, and course modules, a component is needed at this time to allow an instance of a module to dynamically discover the online presence of other module instances. And the offline situation, so that the load balance can be called to the interface of each module. Therefore, the service registry was born, which can help the fitness system to manage and monitor the calls of each module.

In a distributed system, the dependencies between services are complicated, and some services cannot avoid failure. As a result, the remote call threads of other services that depend on these will be blocked, and the entire system service will collapse. The microservice architecture displays a fuse model to ensure the availability of service instances and prevent thread blocking caused by service instance failures. The status of the fuse reflects the availability and robustness of the program and is an important indicator. Here, the fitness system uses Netflix's open source Hystrix as the fuse of the system API interface and uses Hystrix to provide the Hystrix Dashboard component to view the real-time status of the fuse through the interface.

Each module or project will have some configuration files, which represent that a system can dynamically adapt to different environments. In the early days of stand-alone systems, it was simple to modify the configuration file on this node. You only need to find the configuration file, modify it, and restart the service. However, in hundreds of microservice machines, it is often unrealistic to modify a configuration. In order to make modifying the configuration files easier and more intelligent, the system needs to use the service configuration center to manage the configuration files in the system in a unified manner.

The microservice architecture is a distributed architecture, and service units are divided according to business. Microservice systems usually consist of multiple small service units. Because of the large number of service units, the operating complexity of the system will become higher. If the service has errors or exceptions, it is difficult to find the abnormal service. In order to solve this problem, the fitness system needs to implement distributed link tracking, monitor the call sequence and flow direction of each request, to clearly display the path of each service request, so that the problem can be quickly identified.

In the microservice architecture style, large-scale applications are divided into multiple small-scale service systems, which can form their own systems. In other words, these small systems can have their own databases, frameworks, and even languages. The system usually provides RestfulAPI style interfaces called by H5, Android, IOS, WEB pages, and third-party applications. For the microservice system architecture, if the front-end UI interface needs to integrate the data of multiple services, you can rely on the coupled query of the database to obtain the final result, such as a traditional single application teacher. When displaying on the UI, it is usually necessary to display a large amount of data on an interface. This may come from various business modules, so the front-end UI interface must call multiple services.

**Figure 5:**
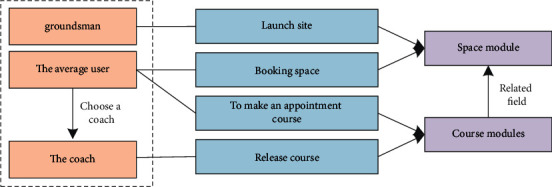
The overall structure of the fitness system.

As shown in Figure 5, this is the relationship diagram between the various modules of the entire fitness system. The venue administrator established a connection with the venue module through the public venue. Ordinary users can establish contact with the venue modules and coaches by making reservations, canceling the venue, and selecting fitness coaches. Because the instructor can publish fitness courses, a connection is established between the route and the route module. Ordinary users can log in to the fitness class and communicate through the appointment route and route module.

### 4.3. Platform Security Design

In the Internet environment, user operations are uncontrollable. In order to prevent illegal and malicious operations by some users, this article will record user login status, empower users, design control access technology, and design security to connect the security of the system at four levels.

(1) User login: the user initiates a login operation on the login interface, and the browser initiates a login request to the gateway. After the gateway receives the login request, it initiates a verification request to the user center. (2) Verify the user request: when a user initiates a service request to the gateway, the gateway will first determine whether the request contains token information, if not, then directly reject the request; if it contains token information, the gateway initiates a verification request to the user center, and the user center queries redis whether there is a corresponding token, if not, notify the gateway to reject the request; if found, notify the gateway to release the request, and forward the request to the corresponding business module for business processing.

When a user initiates a service request, the gateway verifies that the user is a logged-in user and then queries the user center for all the user authority information. The gateway compares whether the request initiated by the user is in the user authority set, and if it is in the user authority set, then let it go The request continues to access the user's business module; if it is not in the user's permission set, then the user is prevented from accessing the system.

On the basis of the control model based on static attributes, risk attributes are introduced, the access control model based on risk attributes is designed, and multiple indicators are selected to evaluate the risk value of the historical behavior of fitness system users. The granularity of access control is refined, and the risk value is used as the value The authorization constraint requested by the user. The size of the risk value has a direct impact on the user's behavioral risk level, thereby indirectly affecting the user's operating authority, that is, the greater the risk value, the higher the risk level, and the more restrictions on the operation. The smaller the risk value, the lower the risk level, there are fewer operating restrictions. All fitness system users who apply for access must go through the policy determination module to access system resources. For fitness system users whose risk value is within a reasonable range, they are provided with access rights, and for users whose behavioral risk value falls within a higher risk range, refuse to provide services to it.

### 4.4. Database Design

**Figure 6:**
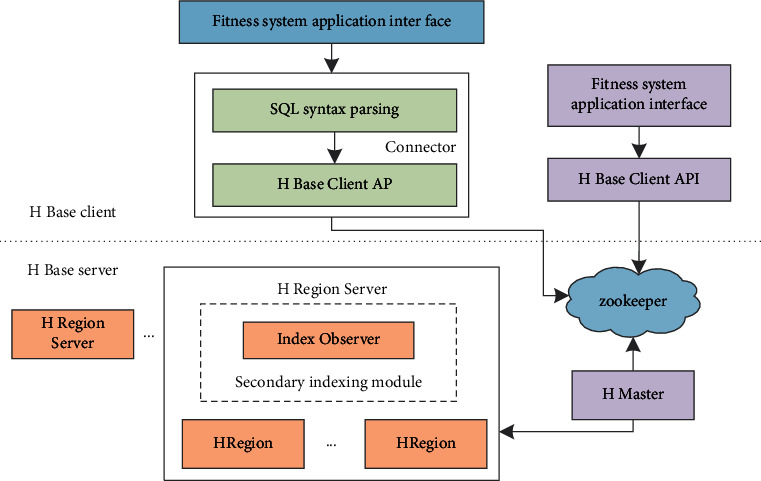
The architecture diagram of the storage management platform of the fitness system.

The overall architecture design of the database is shown in Figure 6.

In order to enable the fitness system storage management platform to support SQL input and support for secondary indexes, the architecture is extended on the original HBase system. The platform consists of two parts, which are HBase clients. Module and HBase server module. In the client module, the fitness system can read and write data on the HBase server through SQL statements or directly read and write data on the HBase server through the HBaseClient API that comes with HBase.

User nodes include ordinary users, coaches, and venue administrators and mainly store basic user information. This information is not always changed and read at the same time. Putting them in the same column family can greatly reduce read and write IO latency. The column family count stores quantity information, such as the number of courses selected, the number of locations ordered, and the number of courses opened. When inserting data in the user table, use the HBaseincrement function to automatically increase the corresponding value. The user nodes see Table 4.

The curriculum table stores the basic information of the curriculum node. The table is also divided into two column families, Classinfo and Count. The information of the course name, start time, end time, coach, and other corresponding infrequent changes is stored under Classinfo. The column family Count stores the number of users in class (as shown in Table 5).

Create a secondary index for querying site information based on user ID for the user site table. The query fields in the secondary index should include user ID, relationship type, and site ID. According to the index interval of each Region, the corresponding secondary index is established respectively. Therefore, the index RowKey for the user to query the venue he reserved is RegionStartKey-userarea-[User ID]0[Venue ID]0. RegionStartKey represents the index range of the Region; userarea represents the name of the user to query their own predetermined index; then the ID of the current user; 0 represents that the user is a normal user; the next is the ID of the venue; the last bit is the status of the relationship, when it is 0, it means that the relationship exists, that is, when querying, this value is always 0. If it is the venue manager who inquiries about the venue information released by him, then just set the relationship type to 1.

Establish a secondary index for querying user information based on the site ID for the user site table. The query fields in the secondary index should include site ID and user ID. According to the index interval of each Region, the corresponding secondary index is established, respectively. Therefore, the index RowKey of the user information of the venue inquired and reserved for the venue is RegionStartKey-areauser-[field ID][user ID]. In the same way, RegionStartKey represents the index interval of the Region; areauser represents the index name of the user information that queries the venue according to the venue; next is the ID information of the venue and the user.

The user course relationship table stores information about the publishing and booking relationship between user courses. The table is divided into two column families: info and time. The column family info stores user ID, course ID, venue ID, user course relationship, and other information. The column family time stores the start and end time of the course (as shown in Table 6).

## 5. Conclusion

This article focuses on the randomness of the initial selection of the center point in the previous literature algorithm and the method of using Min-max in the nonnoise field. There may be a compact selection, easy to fall into local optimization, clustering instability, and eventually may affect the aggregation. Class effect: after research, a second power combination K-means clustering algorithm is proposed. In addition, end-point detection algorithms and speech enhancement algorithms commonly used in the current research field are imported, which provides a theoretical foundation and foundation for tracking algorithm research. This article applies HBase storage technology and microservice framework to the fitness system and implements a national fitness system based on HBase and microservices. The system uses HBase to store the fitness information of the national fitness masses, the opening and use information of the venues, the coaching and evaluation of mass data such as information; using microservice architecture to split the system into user modules, venue modules, and course modules to reduce system integration, improve system maintenance, and build a service system for general users, fitness coaches, and venue managers. It analyzes the functional requirements and nonfunctional requirements of the fitness system, and then analyzes the use cases of the main functions of the system. The function analysis of the system is mainly based on the functions of the general users of the system, fitness trainers, and venue managers. Nonfunctional analysis is mainly to analyze the response speed of the system server, system security, and data integrity. In response to the above demand analysis, this article has carried out a comprehensive design from the system architecture, business architecture, security architecture, and storage architecture.

## Figures and Tables

**Table 1:** F1 value statistical results of end-point detection of different algorithms.

Noise type	Algorithm	Signal-to-noise ratio/dB		
		−5	0	5
Airport	Double Threshold Judgment Method	38.68	53.86	66.57
	Cepstrum Distance Method	26.58	35.04	42.78
	Adaptive Algorithm	66.46	75.78	81.22
Babble	Double Threshold Judgment Method	36.69	53.52	65.15
	Cepstrum Distance Method	28.74	46.37	54.71
	Adaptive Algorithm	69.05	77.91	83.01
Car	Double Threshold Judgment Method	38.90	50.96	65.60
	Cepstrum Distance Method	26.94	47.43	51.22
	Adaptive Algorithm	68.54	76.41	80.37
Exhibition	Double Threshold Judgment Method	36.77	53.58	65.54
	Cepstrum Distance Method	29.24	46.01	53.18
	Adaptive Algorithm	66.89	75.36	82.32
Restaurant	Double Threshold Judgment Method	18.05	36.01	44.79
	Cepstrum Distance Method	59.46	68.61	71.31
	Adaptive Algorithm	27.78	40.51	56.55

**Table 2:** Information of the test data set.

Data set name	Number of objects	Attribute dimension	Number of clusters	Number of objects per cluster
Blood	748	4	2	178570
Parkinsons	195	22	2	14748
Planning	182	12	2	13052
Vertebal	310	6	3	15010060

**Table 3:** Experimental results on the blood data set.

	N-precision	Precision
Algorithm	0.769	0.739
The first document	0.738	0.722
Second document	0.738	0.722
The third document	0.738	0.721
The fourth document	0.738	0.723

**Table 4:** User node table.

Family	Column	Description
Cf1:userinfo	UserName	Username
	PhoneMum	Phone number
	Sex	Gender
	E-mail	Mailbox
	Pwd	Password
	Remark	Remarks
	Fiag	Logout flag
	RoleName	Character
Cf2:count	ClassCount	Number of courses
	OrderCount	Number of venues ordered
	ClazzCount	Number of courses
	GroundCount	Number of release venues

**Table 5:** Course node.

Family	Column	Description
Cf1: classinfo	UserId	Teaching coach
	ClassName	Course name
	StartTime	Starting time
	EndTime	End Time
	Desc	description
	Flag	Delete course logo
Cf2: count	orderCount	Number of students in class

**Table 6:** User course relationship table.

Family	Column	Description
Cf1: info	UserId	User ID
	ClassaId	Course ID
	Relation	User-course relationship
	AreaId	Venue ID
	Flag	status
Cf2:time	StartTime	Scheduled start time
	Endtime	End scheduled time

## Data Availability

The data used to support the ﬁndings of this study are available from the corresponding author upon request.
